# Record DNA: reconceptualising digital records as the future evidence base

**DOI:** 10.1007/s10502-023-09414-w

**Published:** 2023-04-12

**Authors:** Julie McLeod, Elizabeth Lomas

**Affiliations:** 1 Department of Computer and Information Sciences, Northumbria University, Newcastle Upon Tyne, NE2 1XE UK; 2 iSchool, Department of Information Studies, University College London, London, WC1E 6BT UK

**Keywords:** Digital records, Concepts, Data, Evidence

## Abstract

A major issue facing society is the extent to which the usability of the digital evidence base is at risk because, in the digital era, the concept of the record has been eroded. The nature and reality of a record are no longer agreed. Addressing the challenges that the digital presents for managing records and assuring their future usability is not one that records and archives scholars and professionals can tackle alone. This article argues that this is a ‘grand challenge’ which requires a broad range of perspectives and expertise and convergence research to resolve. It discusses findings from an international multidisciplinary research network established to critically explore, through a grounded theory approach, the nature of a digital record and the implications of the digital era for the usability and functionality of the future evidence base. A series of different visions of a digital record emerged alongside a wide-ranging set of research questions that form the basis of an agenda for future collaborative (convergence) research.

## Introduction

Citizens, scholars, governments and organisations rely on records as the evidence base for a wide range of reasons, including informing debate, for law and policymaking, research, development and innovation, for accountability and transparency, conducting business and public inquiries, to ensure our memory and identity creation, and for informing our review of the past. They do so in the understanding that records managers and archivists have a base of theory and practice that enables access to records that are original, authentic, trusted, have provenance and context and are useable through time. However, a major issue facing society is the extent to which the usability of digital records, and as such the digital evidence base, is at risk because the concept of the digital record has been eroded. As professions and disciplines have evolved around the creation and management of digital records, differing understandings of what is meant by a record have emerged.

This article discusses perspectives of a digital record and ideal evidence base that emerged from an international multidisciplinary research network (Record DNA). The network explored the concept of the digital record in relation to identifying the key issues and challenges of ensuring the future usability of the digital evidence base by all stakeholders through time. The data collection took place from 2017 to 2018 with the analysis phase continuing into 2019–2020. The final write up was disrupted by the global pandemic. Whilst some of the outputs of the work have been presented on the network’s website, this article seeks to draw together a full academic analysis of the data, grounding it with the literature. It begins with an overview of relevant extant literature and then presents a series of visions of a digital record developed from the network’s activities, which was informed by a grounded theory approach. Perspectives on the ‘ideal’ digital evidence base are shared and the implications for research needed to tackle the challenge of ensuring its future usability are identified, together with steps towards achieving this.

## Context—wicked problem or grand challenge?

In the physical (paper) world, the nature of records is relatively well understood and familiar (although there remain some points of debate, but in the digital world some contend that the nature of records is shifting. In the digital world the ‘container’, i.e. the file, is no longer the record. The record comprises the granular objects that are scattered yet linked across networks, for example in chains of emails, tweets and text messages, blogs and data. Traditional concepts of a record have understood it as fixed and unique. Some digital technologies have sought to facilitate aspects of the concept of fixity, for example through PDF/A or blockchain (Bralić et al. 2020; Lemieux 2016; Suri and El-Saad 2021). However, the concept of fixity and uniqueness remains conflicted in the digital world where systems automatically generate multiple instantiations or copies of documents across networks (e.g. emails held by both sender and recipient), giving rise to questions about whether there can be such a thing as an ‘original’ record (see for example, Duranti 1989a and 1999; Duranti and MacNeil 1996; Latham 2016; Levy 2000; Lomas 2013; Ostrzenski 2015; Xie 2011; Yeo 2010) and, if so, which is the original (Duranti 1989a, pp 19–20); whether instead we need to speak of the “native format” of digital records, since “the original proper only exists for a nanosecond, if it is ever generated” (Ostrzenski 2015, p 386); and if we “need to replace the binary differentiation of original and copy with a more nuanced understanding of originality” (Yeo 2010, p 106). Not only is the concept of originality, as defined in diplomatic theory, being challenged by the digital but also the concept of preserving digital records. Almost two decades ago the InterPARES 1 Preservation Task Force concluded that “[e]mpirically, it is not possible to preserve an electronic record: it is only possible to preserve the ability to reproduce the record” from that which is stored, and referred to this as “a paradigmatic shift” with “both archival and technological dimensions” (Preservation Task Force Report, InterPARES 1 1999–2001, p 5). As Smit (2017, p 253) notes “[j]ust like installation art, a digital record can never be preserved in such a way that it ‘always stays the same’. The data must be migrated and converted. Data must be added. Formats must be substituted. All these processes must be documented. To keep the record available, it might even be necessary to violate the authenticity of its components: the data." However, if there are no ‘original’ records in the digital space, as we have traditionally conceptualised them, what does this mean for the future evidence base?

Traditionally the concept of fixity (Pearce-Moses 2005) has enabled users to trust records and use them as evidence. However, Levy (2001, p 36) suggests that “all documents—are static *and* changing, fixed *and* fluid” and, from a records continuum perspective, McKemmish (2001, p 335) suggests that “while a record’s content and structure can be seen as fixed, in terms of its contextualisation, a record is ‘always in a process of becoming’”, a notion she had first shared in the early 1990s (McKemmish 1994, p 200). Records are “by their nature not fixed; they are fluid, ever-forming” (Reed 2005, p 128). As Keen (2007, pp 23–4) observed, in the digital world “the record, not the remix, is the anomaly today. The remix is the very nature of the digital”, a view echoed more recently by Acker (2017, p 316) when saying “the very basis of the digital is its flexibility, unfixity, combinations and re-combinations”. Fixity can, therefore, be problematic in a digital world that calls for flexibility and information repurposing (Lomas 2013). The concept is being replaced by uncertainty, mutability and the notions of fluidity (Duranti and Thibodeau 2006) and liquidity (e.g. Bauman 2007; Lomas 2013; McLeod 2008; McLeod and Lomas 2013; Smit et al. 2017).

Digital records, however, do comprise individual elements, from the body of information and metadata through to software and hardware infrastructure components, that link globally to create the presentation of a complete object. This ‘DNA string’ can change, degrade or break over time; alternatively, it can be maintained via migrations, or combined and strengthened through mashups, linked data, analytics, blockchain (including non-fungible tokens), artificial intelligence and other disruptive technologies. In an evidential context, it is important to have exactly the records one requires and not just information on a certain subject. Whilst we live in a world of information overload, reliance on the assumption that we will always have useable contextualised information is risky, particularly when information is increasingly being used as a tool for subversion and attack (Bennett and Livingston 2020; Trace 2022). It is records that aid the delivery of an ‘evidence base’. The existence of an ‘evidence base’ may be understood to have a reliance on the existence, availability, and useability of records. Whilst definitions vary, in essence the ‘evidence base’ is the available body of facts or information indicating whether a belief or proposition is true or valid. Evidence itself is somewhat broader, particularly if viewed through a philosophical lens whereby it may, “consist of such things as experiences, propositions, observation-reports, mental states, states of affairs, and even physiological events, such as the stimulation of one’s sensory surfaces” (The Internet Encyclopedia of Philosophy, 2023). There is a whole philosophical, historical and legal debate, not only on the needs of evidence but also the outcomes of an absence of evidence (Turvey 2008; Kelly 2016). If we are to avoid an absence of useable records, we must ensure we understand the DNA of records and manage them effectively through time. Since our future evidence base will be largely, although not exclusively, digital and multi-media, it is crucially important that it is as reliable, authentic and trusted as the extant physical evidence base, and at least as useable through time, if not more so, by both humans and machines. In addition, in an age of misinformation it must be trustworthy and capable of being interrogated and explained to all stakeholders.

Achieving this is not a trivial goal. We might frame it as a ‘wicked problem’ (as Childs and McLeod (2013) did with managing electronic records) in contrast to a tame one (Rittel and Webber 1973). A wicked problem is one that is ill-defined, essentially unique and a symptom of another problem, which relies on judgment for resolution. It is a problem that is “only re-solved—over and over again” (Rittel and Webber 1973, p 160) and where every ‘solution’ counts and has significant consequences. Alternatively, we might frame it as a ‘grand challenge’ mirroring the change to a positive narrative used by scientists and policymakers this century rather than the previous one (Kaldewey 2018). A grand challenge is one that is “ambitious but achievable” (Kalil 2012, p 3), has potentially global impact, needs considerable research across many disciplines, has well-defined metrics, is realistic to achieve “through creativity and commitment” and, perhaps crucially, can “capture the popular imagination, and thus political support” (Gould 2010, p 64). Some of the most pressing grand challenges are science and technology related, for example the environment, energy, disease and health, with the latter becoming even more visible on a global scale through the Covid-19 coronavirus pandemic. Whilst ensuring the future usability of the digital evidence base may not seem as urgent or significant as these concerns, it is a global challenge that underpins decision-making and everyday societal operation, and usually attracts media attention when a lack of evidence (or failure to use evidence) results in (or contributes to) a major tragedy or incident. In addition, the value of evidence has come to the forefront of public consciousness during the Covid-19 pandemic. Framing it as a grand challenge would “energize” the multiple stakeholders “to develop a sense of the possibilities, an appreciation of the risks, and an urgent commitment to accelerate progress” (Omenn 2006, p 1696).

## Existing perspectives on the concept of a record

An extensive body of archival science literature exists that examines the “contested” (Gilliland 2014, p 171) concept of a record. Over the last quarter of a century this literature has focused on the challenges to the concept brought about by the digital environment. In addition, scholars in other disciplines have contributed their perspectives on records, archives and information (Clarke 2018, p 1–3). For example, in computer, library and information science (Buckland 1997; Levy 2001; Owens 2014), digital forensics (Cohen 2011; Daniel and Daniel 2011), media archaeology, data science, semiotics, genre studies and organisation science (as seen in the book edited by Smit et al. 2017), in law (e.g. Baron and Attfield 2012; Mason and Seng 2017; Paul 2008; Schafer and Mason 2017; Weir and Mason 2017), and in philosophy (Borgmann 1999; Floridi 2002 and 2011). In addition, digital humanities (e.g. Egan 2023) and historians (e.g. Ernst 2012, 2017a, b; Hauswedell et al. 2020; Milligan 2019) have brought their perspective on the records/archives evidence base, including the post-truth context (Vogelmann 2018). It is not possible here to provide a comprehensive, in-depth review of the extant literature (for an example of such a review see Yeo 2007 and 2018). Instead the focus is on a number of key themes that have emerged, in particular the concept of the record per se, the characteristics of a record in the digital world, and the implications for the future digital evidence base.

Traditional views of a record are that they are physical and conceptual objects. It has been claimed that many of the principles and processes from a paper paradigm can and do translate to a digital world. As far back as the 1980s, Duranti (1989a and 1989b) discussed the role of diplomatics in providing a framework for both paper and digital paradigms. She identified seven “necessary and sufficient components” of a digital record (ranging from medium to context) based on diplomatics (“a diplomatic analysis”) (Duranti 2010, p 81), and concluded they “are the same as those of its traditional counterpart, although may manifest themselves in different ways” (p 152). Under her leadership diplomatic analysis has underpinned the InterPARES research (see http://www.interpares.org/). Other early work on electronic records highlighted their logical nature. Bearman (1996 p 196) asserted that, unlike physical (paper) records, digital records “are only logical things” which “can be physically housed in many places but seen together. They can appear to have different arrangements depending upon the views accorded to their users. In other words, the properties of logical things are associated with them through formal, defined, logical relations”. Indeed, one of the structural principles of the records continuum model is a focus on records as logical rather than physical entities, irrespective of their (paper or electronic) form (Upward 1996). Despite Bearman’s claim that digital records are “only” logical he clearly does acknowledge their physicality or materiality. These characteristics are recognised by Thibodeau who asserts that digital records, or digital (information) objects, have three types of properties—physical, logical and conceptual—which “can be significantly different” (Thibodeau 2002, p 2). Again, it is the addition of logical properties (the recognition and processing of the object by software) that is the key difference to traditional views of a record. InterPARES 2 examined specifically the “interactive, experiential and dynamic systems that do not necessarily produce or keep anything that corresponds to traditional records” (Duranti and Thibodeau 2006). In this context, the project developed the idea of a “chain of preservation” as necessary as opposed to the chain of custody that could exist in paper paradigms (Eastwood et al. 2008). In 2017, Voutssas mapped the concept of the digital record drawing on concepts of archival science, in particular diplomatics, and mostly, though not exclusively, through the phases of the InterPARES research programme (http://www.interpares.org/). His ontology identified a digital record as comprising of intellectual components and digital components, where the same intellectual components may exist in digital components and the latter always residing on a physical component stating, “a digital record may be seen either conceptually as a set of formal parts having certain characteristics and relationships which ideally shape the record—named “Intellectual Components”-, or a digital record can be seen computationally as a set of digital information objects—named “Digital Components”—which compose the digital record residing on a physical medium or support” (Voutssas 2017). Acker (2017, p 297) believes materiality is more than the physical form, it is “the processes of records’ production, transmission and storage” and extends to “the systems, practice, and social institutions that are built up around artefacts” (p 298). The importance of understanding the materiality of the digital, if we are to “take care” of it, is signaled by Floridi (Glaudemans et al. 2017a, p 309) in noting that it “is of a different type than the materiality of the analogue.” He illustrates this with the example of blockchain technology and the potential environmental implications of its energy consumption if widely adopted.

Yeo (2007, 2008 and 2018) presents a very different perspective on the concept of a record, viewing records not as information objects or containers but as a type of ‘representation’. Initially he characterised records as “persistent representations of activities, created by participants or observers of those activities or by their authorized proxies” (Yeo 2007, p 337) but later extended this “to encompass not just activities, but steps, processes, functions, and other such phenomena”, resulting in the characterisation of records as “persistent representations of activities or other occurrents, created by participants or observers of those occurrents or by their proor sets of such representations representing particular occurrents” (Yeo 2008, p 136). He explains that the term ‘occurrents’ (borrowed from philosophy) is being used here as the collective noun for “concepts such as function, process, activity, transaction, and event” (Yeo 2008, p 136). Activities encompass “the full range of deeds and actions humans undertake” and those “performed by mechanical devices on human instructions” (Yeo 2007, p 337). Yeo’s focus on the performative characteristic of records, linked to acts and with both social and informational roles, is rooted in speech act theory rather than diplomatics (Yeo 2007 and 2018) and has been described as a “proper, contemporary definition of records” (Glaudemans et al. 2017a, p307).

In 2005, Gilliland wrote “[T]he record remains a problematic construct even within the archival community. Within the U.S., there is insufficient common understanding of the nature of the record and how the record as a construct might be operationalised in digital environments, such as distributed and multiprovenancial databases where there is often not a readily discernible physical information object that corresponds to paper notions of a record” (Gilliland 2005, p 221). However, she later suggested a consensus can be drawn from the varying definitions summarising it as, “a record is always associated with some action, transaction, or event; a record can be a product, a by-product of, or even an agent or actor in an action, transaction, or event; and a record includes, at a minimum, a definable set of metadata that serves to provide contextual and other forms of evidence about that action, transaction, or event” (Gilliland 2014, p 176).

Some of these perspectives have informed and/or been informed by the international records management standard which identifies the qualities of an ‘authoritative record’ as delivering authenticity, integrity, reliability and usability (ISO 15489-1: 2016s 4 and s 5.2.2; ISO 30300: 2020s 3).

More recent perspectives emphasise the ‘dramatic’ change in the nature of a record due to the digital environment and challenge even this (base) level of consensus. Smit (2017, p 263) suggests digital records are now “innumerable objects of information” which fit the five properties of what philosopher Timothy Morton (2012) terms ‘hyperobjects’, i.e. “objects that exist beyond the possibility of humans to perceive”, comprehend or “grasp… in full” (Smit 2017, p 255). He says “[T]he resemblance with digital records is striking. They are by their very nature contextual. If they are managed well they transcend our own timescale. Digital data are so vast that it is hardly possible to get a grip on their whereabouts. They are not bound to specific locations. And their use is time and location independent. And records are sticky: they will influence you in any time and in any place. Digital records also have the tendency to withdraw: once they have been made manifest, they tend to hide again (in the cloud for example), like an octopus” (Smit 2017, p 256). Within this context, the aspects which make a record appear or manifest as understandable are complicated by the components including, but not limited to, tools such as the algorithm (e.g. Andresen 2020; Bunn 2020). However, Ketelaar (Glaudemans et al. 2017b, p 303) dismisses the concept of hyperobject and takes a more pragmatic approach arguing that a record can be defined to suit needs and, using the capture of PowerPoint presentations into a recordkeeping system as an example, states that “what defines ‘record’ here is the policy; it is not a question of (archival) theory”.

Both Bearman (1994) and McKemmish (1994) have discussed the notion of ‘when’ rather than ‘what’ is a record, Bearman relating this to the transaction (i.e. the sharing with another person or a machine and its accessibility) and McKemmish to the crossing of boundaries. Acker also prefers to consider ‘when’ is a record, arguing that “a record’s mediation through time and space, its mobility and its perdurance as archival infrastructure are the ultimate factors in determining enduring value. Analysing how a record moves through networks, and asking when a record is becoming, and how and where it persists is a more productive method for understanding the nature and effects of infrastructure and ultimately how electronic evidence is socially constituted with technologies” (Acker 2017, p 316). In data science, data quality, provenance and lineage have become critical concepts for systems delivery (Doerr et al. 2011). Despite this focus on data,’what is a record?’ was the title of the opening conference of a more recent network created by social scientists at Aalborg University, Denmark on the digitisation and the future of archives (IRFD 2019), and ‘rethinking the record’ was one of five core research themes of The National Archives of the UK (The National Archives 2018). It is implicit in their current core research themes (The National Archives 2019).

Extant literature, mostly situated in the records and archival science domain but increasingly in other disciplines, reveals perspectives on the nature of the digital record ranging from the granular, to the object-oriented, performative and data processed hyperobject, and the policy-based definition. The level of debate in this arena indicates the significance of understanding digital records and their value in the context of the delivery of an evidence base.

## Records and evidence

Records have been a key resource for the delivery of evidence for thousands of years and within this context fixity and standardized formats has been a part of the assurance of the provision of an ‘authoritative’ record for evidential purposes both in terms of immediate and longer-term delivery. We see this in the physical manifestations of records created as clay tablets, parchment rolls or through wooden tally sticks. The tie between recordkeeping systems and the evidential needs of governments and the legislature became increasingly overt and defined through the ages with the growth of complex recordkeeping systems (such as the Roman Tabularium and England’s medieval registries) (see Clanchy 1987; Yeo 2021) and legal requirements, including admissibility rules (Padoa-Schioppa 2017). The term ‘evidence’ in the English language is explicitly interwoven in its development, although not synonymous, with the ability to produce a record in an acceptable format for legal purposes. The complete version of the Oxford English Dictionary (2023) cites this link with examples from the fourteenth century onwards of legal cases being progressed through the production of evidence in the form of particular fixed record formats from charters through to title deeds. However, equally it notes the significance of oral records and testimony and broadens its definitions of evidence to “information (in the form of personal or documented testimony or the production of material objects), tending or used to establish facts.” Within the context of legal evidence, it has long been accepted that all evidence is not equally reliable and as such needs to be weighted taking into account the use and contexts surrounding it (Krauss and Scurich 2013).

The need to evaluate and weigh records as evidence of the past is well understood by historians and archivists (Burke 1997) with Spragge (1992 p.212) making the case for the value and care of archival records, asserting “the past is a tenuous shadowey reality, half misunderstood, half forgotten, For us to live with it, it must be accessible, physically and mentally, and must be valued and cared for.” Within this context, historians have wanted to provide uncontested facts as part of the evidence base. However, increasingly it has been recognised that there is the potential for multiple truths (Lynch 2009; Wright 1992), albeit that some events are incontrovertible, as established in the 2020 legal case Irving v Penguin Books Ltd which held Irving had misrepresented events in terms of Nazi actions denying the Holocaust for personal ideological reasons. In contrast to historians, scientists in the nineteenth and twentieth centuries for far longer pursued certainty and sought to develop an evidence base of research knowledge, which could be relied upon. This concept is defined in the Frascati Manual as the development of “a stock of knowledge” (OECD 2015). The twentieth century saw an increasing debate about the credibility and value of a range of research evidence dependent upon a number of factors influencing its generation and capture. These debates centred around distinct approaches to evidence generation with positivists arguing that only the most scientifically reproducible approaches to the generation of knowledge could truly produce evidence. Others argued for new approaches to evidence generation which take into account more contextual and human considerations, making the case for qualitative approaches to evidence generation. Filstead discusses these arguments representing “fundamentally different epistemological frameworks for conceptualising the nature of knowing, social reality, and procedures for comprehending those phenomena” (Filstead 1979 p.45). Today the values of differing approaches to evidence generation in research contexts are more generally accepted albeit that critiques of different approaches may still be made. Within the UK Government there has been a drive to generate policy and regulation based on a ‘rigorous evidence base’. This ambition was expressed in 2015 with the start of the production of Areas of Research Interest (see https://www.gov.uk/government/collections/areas-of-research-interest). This has been linked to UK’s The National Archives’ ‘rethinking the record’ (The National Archives 2018).

More generally, in a UK government and policy-making context, a number of public inquiries have foregrounded the need for records to provide evidence to support inquiries, identifying problems with digital ways of working, a lack of the survival of evidence, and issues with understanding records and evidence in their creation contexts. These have included, but not been limited to, the Hutton Inquiry (see Moss 2005) and the Hillsborough Inquiry (see Cooper and Lapsley 2021). Internationally there are been similar findings in inquiries, for example the Royal Commission into Institutional Responses to Child Sex Abuse in Australia found similar failings in its evidence reviews and made specific recordkeeping recommendations (see https://www.childabuseroyalcommission.gov.au/recordkeeping-and-information-sharing).

Within each of these contexts, the challenge of weighing electronic records and their evidential value has been increasingly explored and regulated for. Within a legal context, in the latter part of the twentieth century, weighting has incorporated considerations of electronic records and their contexts as is seen in the British Standard (BS 10008) on legal admissibility for electronic records which evolved from the 1990s onwards to its current 2020 (BS 10008: 2020) format and in a USA context the Sedona Principles (The Sedona Conference 2018) currently on their third published version which has been the product of evolution through 19 conferences. Within historical contexts, the need for specific skillsets to understand digital evidence, records and archival considerations has developed new disciplines in terms of the digital humanities and computational archival science (Terras et al 2013; Hedges et al 2022). In each of these contexts, the implications for the future of the digital evidence base and its position to individuals and society are still evolving.

## Research investigation

In the context of today’s digital reality the research aim was to identify the key issues and challenges of ensuring the future usability of the digital evidence base by all stakeholders through time. Specifically, it sought to a) problematise the current concepts of the digital record and work towards a reconceptualisation that more adequately facilitates its management; and b) identify what research is needed to address the challenges and ultimately realise good practice solutions to ensure the future digital evidence base can be provided to users in reliable, trusted and useable formats they can interrogate. The research was designed by the Principal Investigator (PI) (with a records management, information science and systems background) and the Co-Investigator (with an archives records management, cyber security and information rights law background). A facilitator (with an ICT background) and four cross disciplinary advisors (with archival science, history, law, records management and technology backgrounds) contributed to the detailed design of its implementation. In an effort to broaden the coverage of expertise and viewpoints of those with a vested interest or relevant knowledge, an international multidisciplinary research network was established (Record DNA). It brought together practitioners, academics and other stakeholders from multiple disciplines with wide ranging expertise to explore how the digital has put the traditional concept of the record at risk and work towards a new concept of the digital record. They ranged from creators to users, and included policy and decision makers, archivists, IT professionals, lawyers, librarians, records professionals, as well as academics and researchers from business, computer science, data science, digital forensics experts, information science, history and the broader arts and humanities.

The researchers acknowledged that they and the advisory steering group had the potential to influence the research. The National Archives in the UK were invested in the value of the research which aligned to its research priorities and linked to broader UK Government concerns related to the survival of digital records linked to evidential needs. However, the intention was to reach out to an international audience and a range of contexts, taking account of a broad range of stakeholder perspectives. The PI and CI viewed the research through an interpretivist lens but there were no prior assumptions about the findings and a grounded theory approach was taken in order to grow the research from the bottom up (i.e. to be inductive) and to thus minimise bias as far as was possible. The term ‘Record DNA’ was chosen as a project name to convey the complexity that resides around the reality of a record and, in addition, to provide a research identity for drawing in research network participants. This was not fully defined for participants as the intention was to open up rather than confine conversations around how evidence and records are created, managed and manifested through time. For some this term was then connected to biological considerations but the researchers did not seek to reinforce these interpretations rather to consider them as part of the research data as a whole. Useability was intentionally highlighted in the research aim with other concepts, such as reliability and trust picked up and highlighted too. Useability of a record is defined within ISO 15489 (2016 p.5) as a record, “that can be located, retrieved, presented and interpreted within a time period deemed reasonable by stakeholders”. The research team and advisors deemed that this term had the potential to bring in wider concepts and considerations of the stakeholders’ needs and contexts of useage. Reliability, as highlighted too, is defined in ISO 15489 in relation to records, whereby a record’s “contents can be trusted as a full and accurate record representation of the transactions, activities or facts to which they attest, and which can be depended upon in the course of subsequent transactions or activities.” A purposeful decision was made not to mirror all the record components defined within the international records management standard developed by recordkeepers (ISO 15489-1: 2016s 4 and s 5.2.2; ISO 30300: 2020s 3). However, reliability was specifically selected as having become increasingly debated in data science and legal contexts in relation to useability. In addition, trust was selected as a much debated term across disciplines but equally one which is open to wide public understanding and much valued in all contexts. The research intention was to rethink critical concepts with a wider range of stakeholders and to allow for new ideas to emerge. As such care was taken not to provide potentially limiting definitions for any concept selected. The selected concepts enabled different disciplines and professions to come to and open up the debate in a range of contexts. The intention was not to be limited to these terms; the research was further framed to allow new and old definitions to emerge and be included.

The bottom-up research approach adopted was informed by second generation constructivist grounded theory (Strauss and Corbin 1998; Charmaz 2006; Morse et al. 2009; Bunn 2017). Mansourian highlights that, “although GT [grounded theory] is a well‐established methodology, it is an approach to research rather than a detailed research method” (Mansourian 2006, p.387). As observed by Bunn, grounded theory, “can enable new theoretical understandings to emerge” as it does not rely on building upon prior empirical understandings (Bunn 2011, p 41). In 2017, she developed her perspectives stating, “the ideas of theoretical sensitivity and of open-ness, start from no other point than the selection of an area of interest and a desire to work out the main concern of the participants in that area and how they (the participants) resolve it” (Bunn 2017, p 523). This approach enabled a focus for the research to be set, but then an inductive building of understanding with different stakeholders sharing their perspectives without reference to a past as the starting point. It was structured through a series of events designed to bring together the range of stakeholders, either face-to-face at workshops or virtually in crowdsourcing activities, enabling participation from any community and any location worldwide (see Record DNA). Each activity built upon the preceding one. The respective order was:

- Workshop 1 in London with purposefully selected participants
- A wiki survey crowdsourcing activity open online globally
- Workshop 2 in Newcastle with purposefully selected participants
- An online survey open globally

For the UK-based workshops, participants were purposefully selected to ensure that there was representation from across disciplines. The invited participants were suggested and agreed between the two researchers and the advisory group members. At each workshop, 30 participants were invited with 23 (workshop 1) and 29 (workshop 2) attending. A total of eight people attended from outside the UK. Participants were sought from across a range of academic disciplines and professional practice positions. In both workshops a range of paper-based activities were undertaken with key discussions being recorded. At each workshop, participants were divided into separate groups with the intention being that groups would contain individuals with differing backgrounds. In workshop 1 the groups were mixed up during the activities. In Workshop 2 the groups stayed the same throughout the session. The expertise/backgrounds of the participants at the two workshops are indicated in Tables 1 and 2 below. It is to be noted that these are researcher-applied labels based on known roles; each participant might differently describe their expertise and, in particular, their longer-term background knowledge.

**Table 1:** Workshop 1—expertise/backgrounds of participants

Archives and records management	Environmental science and technology	Journalism	Records management
Computational archival science	Genealogy and history	Law—human rights	Science
Computer science	History	Law—information rights	Social Science
Contemporary history	ICT	Law-e-discovery	UK government inquiry management
Digital art	Information governance	Physics and research data management	
Digital preservation		Policy-central government

**Table 2:** Workshop 2—expertise/backgrounds of participants

Group 1	Group 2	Group 3	Group 4	Group 5
Digital preservation	Computer science	Computer science	Digital forensics	Computational archival science
Systems and records management	Internet archival management	Law and information rights	Digital preservation	Blockchain and product/operations management
Open data and records management	Cyber security	Public sector systems delivery	Data science	Systems and digital preservation
Philosophy	Digital humanities	Digital preservation	Philosophy	Policymaking and research data management
History	Records management	Computational archival science	Digital humanities	Maths and computer science
Archival Science		Information governance	Archival Science	Digital curation

The wiki survey and traditional survey were cascaded by the advisory group and workshop participants. Responses to the online approaches were received from all inhabited continents.

In line with the grounded theory approach, the initial activity in the first workshop began with a ‘silent start’ to break group thinking and align to the bottom-up inductive approach. Individuals were asked to think about the benefits of delivering the aim of the network, with participants considering what they wanted from the ‘digital evidence base’. These ideas were captured on post-it notes. All of the post-it notes were then reviewed and discussed considering any synergies and patterns. These were then rated in terms of ultimate goal, primary goal and benefits. Some consideration was given as to what was essential in this context. Following this opening activity, the problem and potential solution spaces were then explored, thinking about the DNA of a digital record both in time and scale. A Triz (theory of inventive problem solving) semi-structured approach to problem solving (Triz https://en.wikipedia.org/wiki/TRIZ) called nine box thinking, or thinking in time and scale, was then adopted (Silverstein and Samuel 2012, pp. 57–63). Participants were asked to capture all the problems and issues that currently exist around the DNA of a digital record in a 3 × 3 matrix (9 boxes) representing time (horizontal axis) and scale (vertical axis). In this instance, time was (1) before a record is created, (2) immediately after it has been created and (3) its existence thereafter; scale was (1) the record, (2) the system(s) and (3) the context/environment beyond). To open up the interpretation of these prompts, analogies from nature were used to inspire alternative ideas of an information ecosystem. This enabled some debate about where different components of a record ecosystem sit. Having built this system perspective, problems around these perspectives were then identified through discussion. The second step involved capturing on a new sheet all the possible solutions for solving these problems with similar scale and time criteria. A report was produced from this workshop which was shared with the second workshop.

A crowdsourcing activity, in the form of a wiki survey, was then used to ask what the components of a digital evidential record are and then to rank these components in terms of significance. The link for the wiki survey was snowballed through academic networks and social media internationally. At this stage no framework was placed on the components. Ultimately, all the components produced as part of the crowdsourcing activity were taken forward to a second workshop where groups of participants critically explored in greater detail their meaning and value. Each group decided which components from the wiki survey, if any, to include in their vision of a record and added further ones they felt were either missing, needed to be refined or described differently. They grouped the components into themes and then linked the themes. They were then asked to develop a vision of the ‘ideal’ useable digital evidence base. This began by writing words or pictures, individually and in silence, on post-it notes that described the ‘ideal’ useable digital evidence base (i.e. what it looks like) from their perspective. Participants were encouraged to include emotional adjectives as well as objective characteristics. They then grouped the post-it notes before discussing and naming the groups of words/pictures.

The final step was to consider where we are now and what research and practice is needed to deliver the vision of the ‘ideal’ (from their perspectives) useable digital evidence base. Workshop participants were asked to identify any research questions that still need to be answered or studied. They selected the two most urgent or interesting ones and expanded them by identifying the underpinning objectives and knowledge/skills/roles needed to tackle them. The complete list of research questions was grouped into 30 themes and a global online survey was used to ask people to rank their importance on a five-point scale where 1 was an interesting area of research for the longer term, 3 was a high priority with relevance across disciplines and/or domains of practice, and 5 an international research priority. Although 195 people started the survey, this question was only answered by 47 people. A number of people indicated that it was extremely difficult to prioritise the themes either because the choice was overwhelming or because they were all important.

It is important to note that the range of tools used to prompt contributions was intended to aid reach to a wide number of participants from across disciplines and to provoke responses in new ways to enrich the data collection. The various tools gathered data through mechanisms which, in some cases, enabled self-coding, for example where participants grouped and themed responses. In other instances, the researchers analysed responses.

Particular limitations of the approach were that the research was undertaken in English only and all events were held in the UK, although travel for some overseas participants was funded. Whilst responses to the online surveys were received from all inhabited continents it is acknowledged that the research is largely representative of Western perspectives on records. The intention of the title (incorporating Record DNA) was to convey complexity however, it is accepted that this may have been perceived by some as an object orientated approach with potentially biological links.

## Findings

The research moved the understanding of a digital record away from the limitations of conceptualising a digital record as an ‘object’ towards developing a conceptual framework based on its DNA—its granular components including its content and context—as it resides across software, systems and networks. The outcomes of the network’s activities were different visions of a digital record. No consensus was reached but rather visions to prompt further discussion and focus on this debate. In addition, visualisations of the ‘ideal’ digital evidence base, taking into account societal considerations (such as the way we create, share and manage information as evidence for the benefit of the society), alongside key challenges for ensuring the future usability of the digital records evidence base and a research agenda for addressing those challenges were developed. The outputs from each activity were published on the network blog and used to develop a series of infographic outputs aimed at different audiences and stakeholders and for different purposes (see Record DNA).

### Visions and perspectives of a digital record

The global crowdsourcing activity identified 89 different DNA suggested components of a digital record. 48 of these were distinct component suggestions. Some suggestions were properties or qualities of a digital record rather than elements (e.g. authenticity or provenance, accuracy and completeness) with a number of the suggestions linking concepts and components, for example, “demonstration of the authenticity, integrity and provenance of a record over time” or simply stating a standard such as Dublin Core. Systems considerations were reviewed, including software and hardware, as well as ideas that moved the record away from object orientated understandings. As such, the components did identify new intrinsic and extrinsic elements. The components took into consideration new forms of recordkeeping, including biological computational science with cells storing data and power sources being provided by biological sources. These ideas had emerged in the first workshop and, within this context, consideration around environmental concerns were articulated. The connection to people and society was noted and perspectives were shared from different disciplines. A genealogist noted that from their perspective name and date of birth were the critical components of a record although another genealogist countered this with it being dependent on the question asked. A historian noted availability as a key record quality and from a legal perspective the value of an intellectual property mark and ownership rights were added. 7982 ranking votes were received which ranked the most important concepts. They were deemed to be metadata followed by content. (Many more people voted as part of the ranking exercise than contributed DNA components).

Analysing the components further, they could be divided into three categories—traditional components, new interpretations of traditional components and new components from a recordkeeping perspective—although the blurring of boundaries needs to be noted. Traditional components ranged from, content, context, and metadata relating the record to the context of its creation (e.g. creator, creation time, related records, receivers) and the history of the record over its life, to open source code, storage device and software. New interpretations of traditional components included concepts such as ‘audibility’. This emphasised the value of oral records but also the consideration that records needed to be heard in new ways with the potential for oral records to become favoured over textual records as voice recognition capabilities increase. In addition, the concept that technology and evidence are not neutral considerations and that recordness resides partly in the hands of the creator and user was stated (Ellul 1990; Caswell 2014; Winn 2017). It was noted here that there are increasingly new forms of knowledge organisation and empowerment, or subversion, emerging all the time, such as the application of a hashtag or the insertion of a chatbot comment. New ideas for the discipline of archival science and records management were ideas around noology where technology and ideology can be navigated together.

**Fig. 1:**
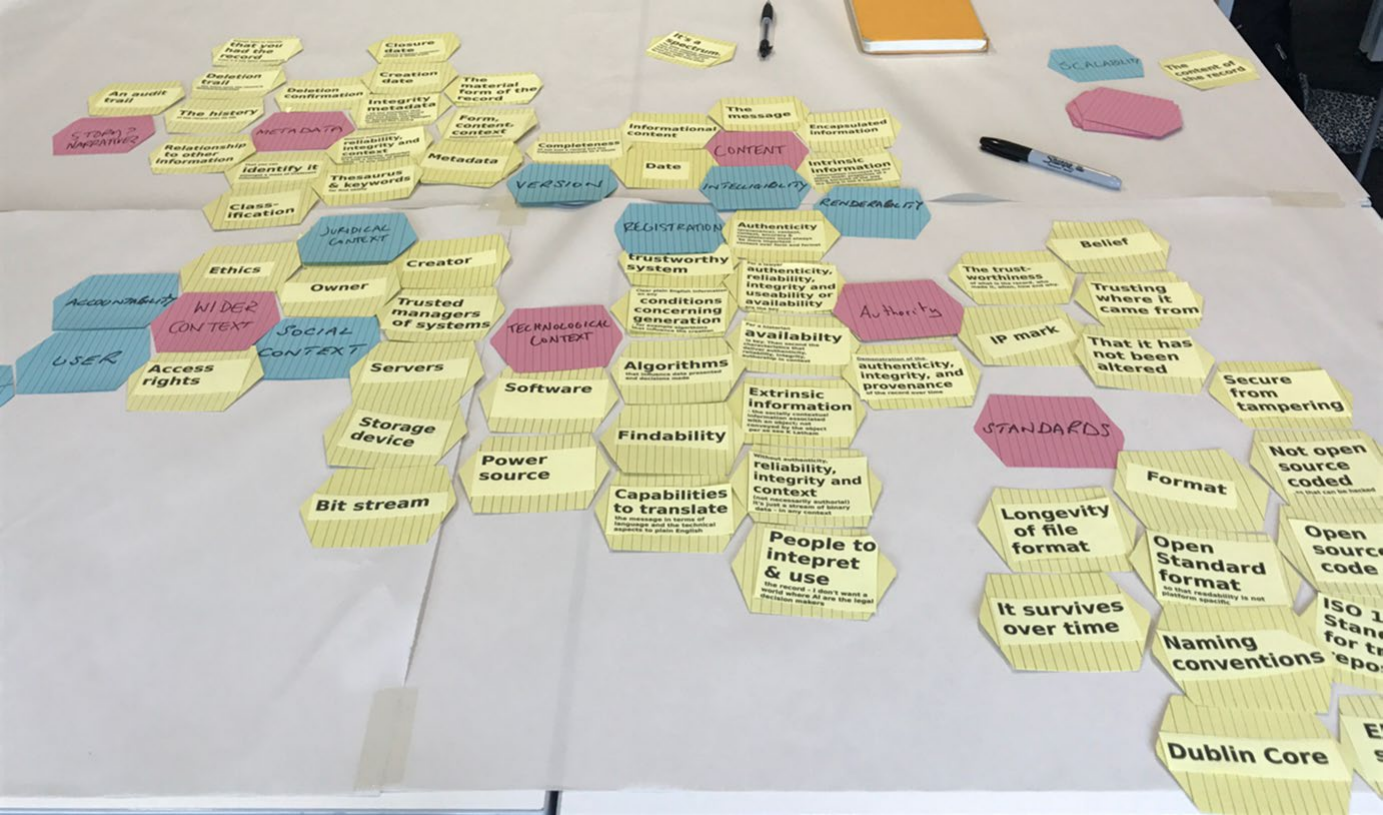
A granular vision of the DNA of a digital record

**Fig. 2:**
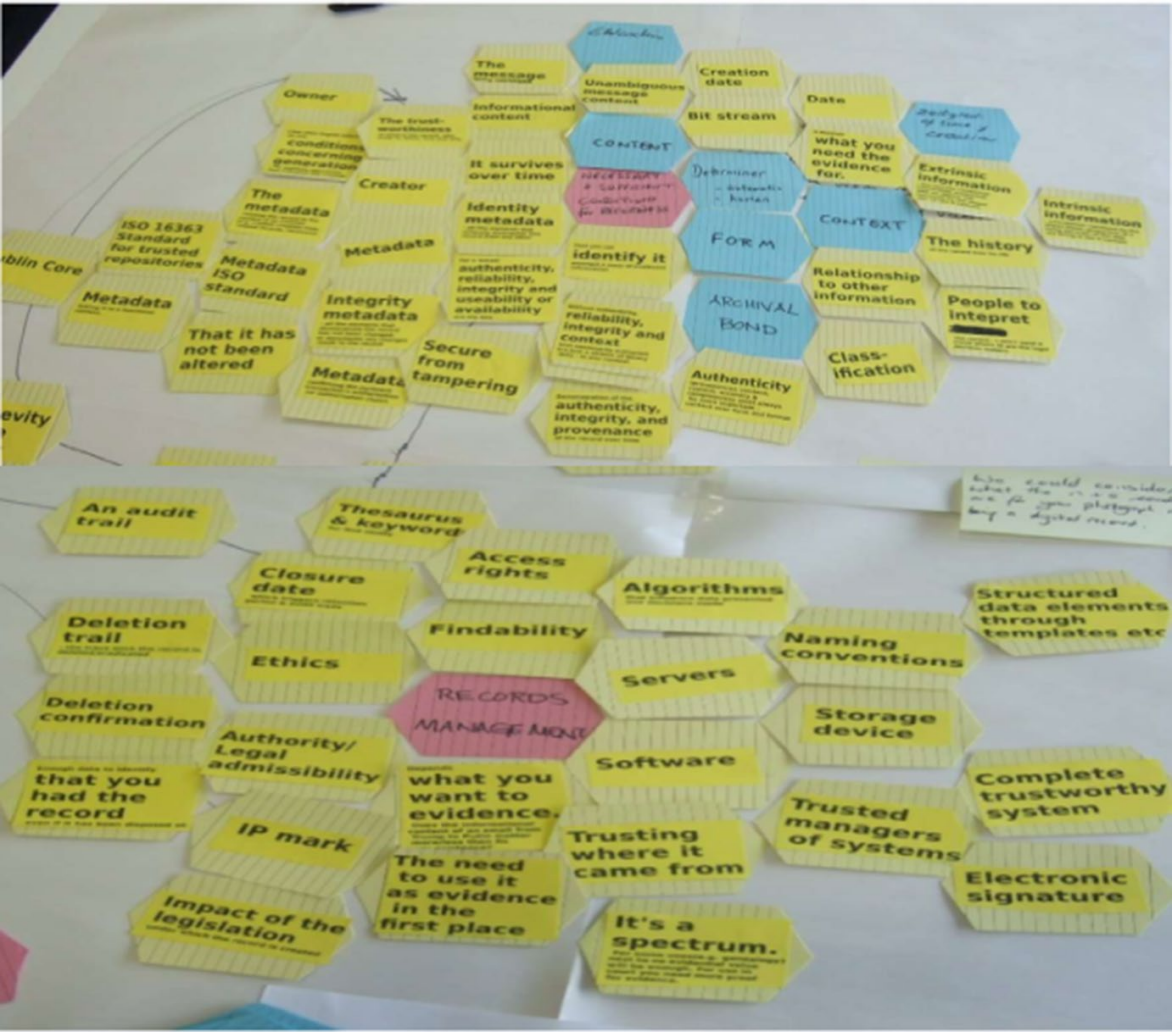
A granular vision of the DNA of a digital record

**Fig. 3:**
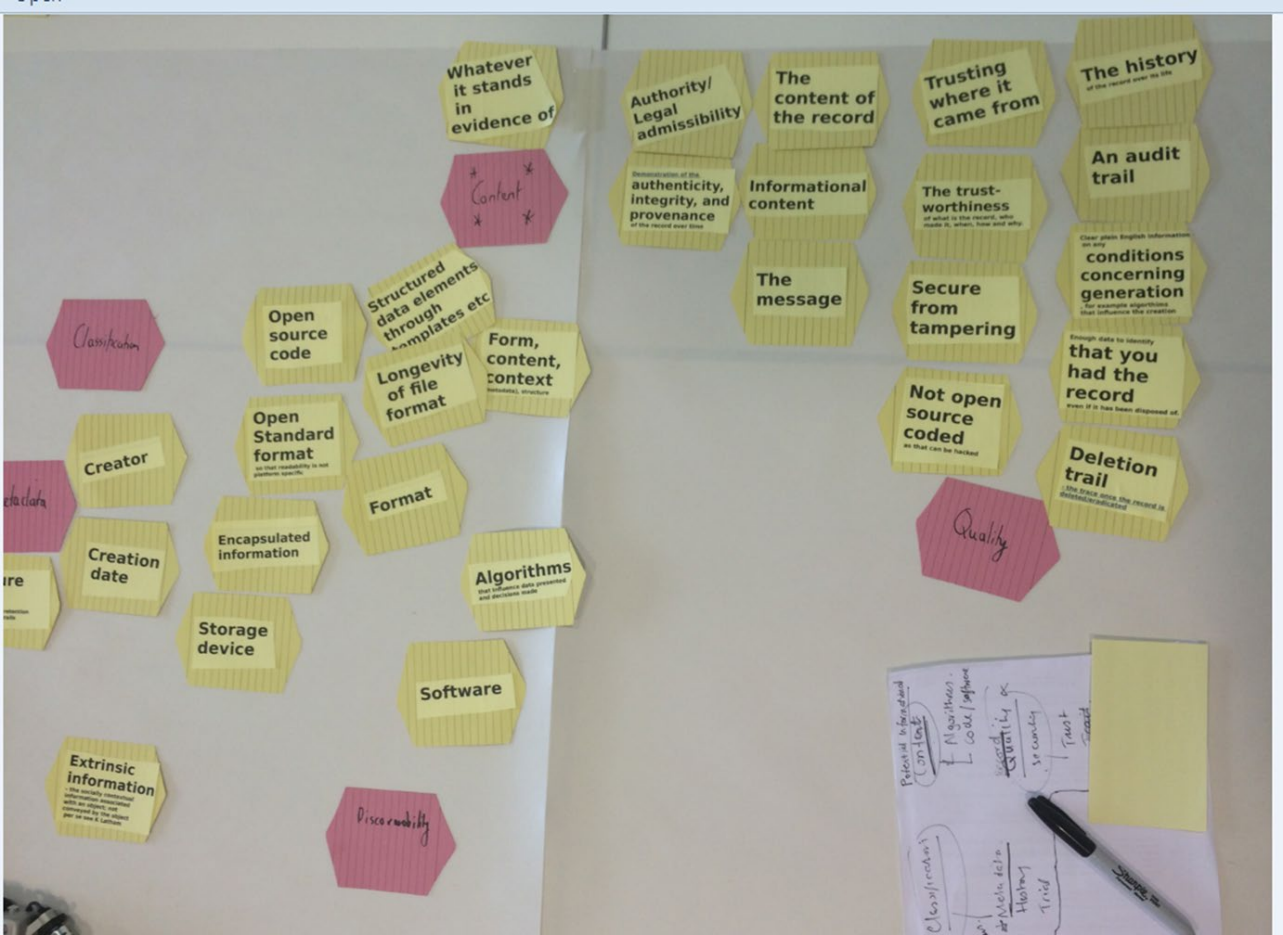
A vision of the DNA of a digital record focusing on content

**Fig. 4:**
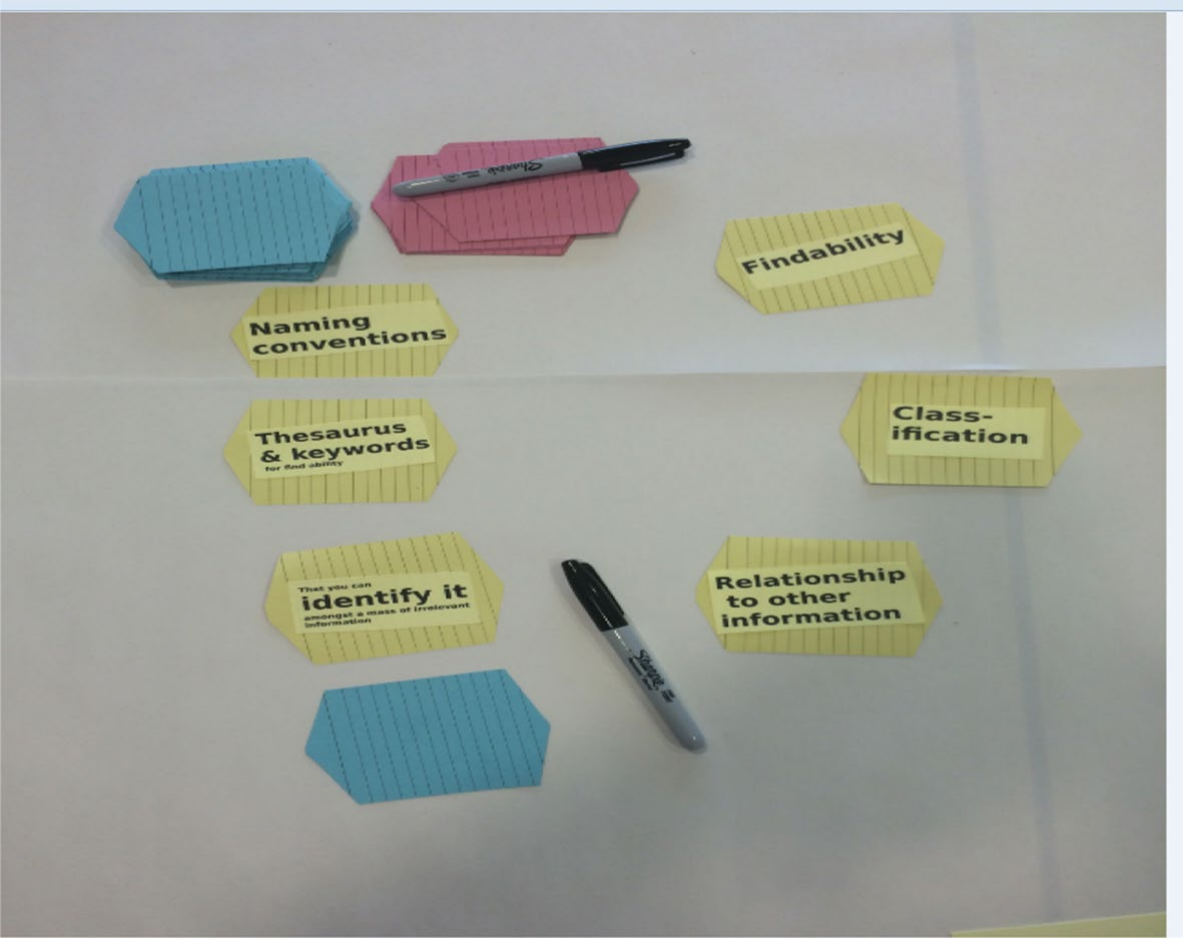
A vision of the DNA of a digital record focusing on information

**Fig. 5:**
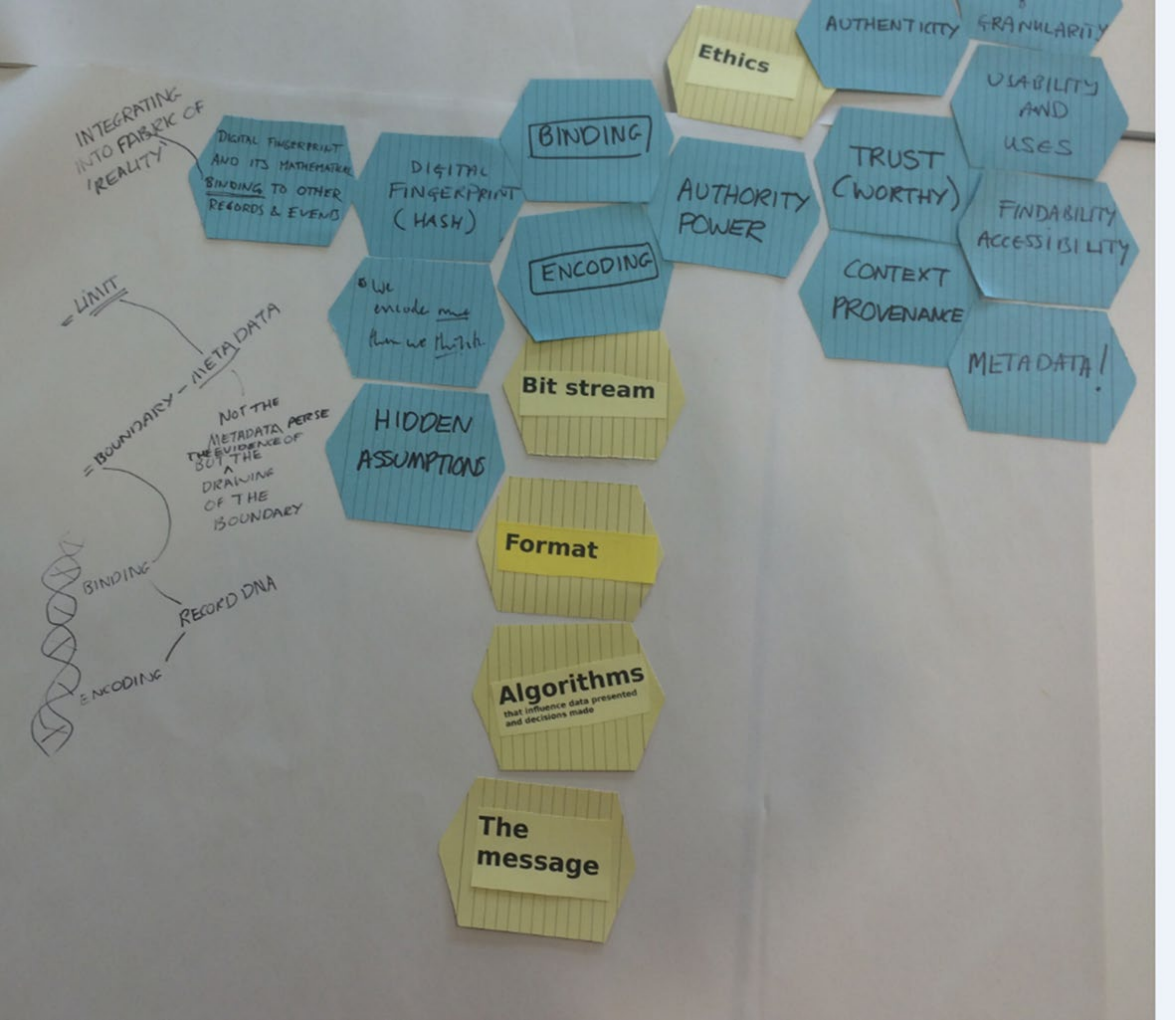
An alternative vision of the DNA of a digital record

The visions of a digital record created at the second workshop, starting from these components, were quite varied. Figures 1, 2, 3, 4, 5 evidence snapshots of these visions as works in progress. They were supplemented with recorded discussions explaining the visions. In terms of the visions, some participants brought in paper-based thinking as valid in certain areas whilst others entirely re-envisaged a digital record. It was notable that some groups built up their visions from the crowdsource components which they then supplemented (Figs. 1 and 2) whilst other groups started with these and worked from more granular visions to stripped back altered perspectives (Figs. 3, 4 and 5).

Figures 1 and 2 both comprise some traditional record components including software, hardware and process considerations (e.g. content, bitstream, structure, metadata, standards and processes). However, they both bring in contextual components, such as ethics, power and trust. In Fig. 1, the role of the human in the loop as the ‘trusted manager’ is more evident with the significance of human ‘belief’ standing in relation to the record. This was reinforced by the captured discussions. Other groups captured alternative visions. Figure 3 identifies that a record is simply ‘content’ i.e. it only matters what information it conveys, although around this concept, was the idea that it has to be managed in whatever form content manifests, including the ideas around biological computational science. This idea resonated with ideas around content management systems. Key at this new frontier was data quality, underpinned with metadata and access through discovery, and dependent upon device and storage. This is perhaps a more ICT perspective and it was suggested that different professions may have different visions. It is notable here that records management was perceived as having a role in managing this content through time albeit perhaps through more technological rather than human interventions. Nevertheless, the place of the human as ‘individual’ and ‘society’ was evident and an important part of the discussion about why the content did matter. Figure 4 shows the group focused on the concept of information (as opposed to content albeit this was aligned), producing a simple loop around what matters to ensure the access to that information through time. Within this context, the boundaries between ‘content’, ‘information’ and ‘recordness’ were discussed and noted by some participants as not important, with information automatically delivering a record. The critical question then discussed was its capacity to act as ‘evidence’ linked to further questions about ‘for whom’ and ‘for what’.

For many the record had altered significantly in the digital age. The vision in Fig. 2 highlights the bitstream and encoding as key components of the record, with what could be a range of attributes connecting these together to present a record. Algorithms and potential other digital code influence the record and, as such, there are hidden assumptions driving its form and creation. Each time the record is generated it may take a new form and alter slightly for each new audience; as such it may then be unique and have some identification. This concept was mentioned in relation to the vision created in Fig. 5, discussed as a fingerprint that aligns to and evidences this manifestation—the digital fingerprint. When it is reborn it is a new record or ‘wash’. The wash may sanitise and alter the record. In this context there were again discussions on biological computing, as well as biometrics, with human data in digital forms becoming embedded. In all visions, the context and provenance of the record influence its form and use through time. In this discussion data provenance and data lineage were concepts introduced by the technology participants as critical ways of knowing. It was observed that USA technology companies sweep data to profile and make assumptions about the creator’s suspected location and nationality, in order to determine their personal data legislative rights. These are the concerns of the data scientist in terms of determining data governance, provenance and lineage, providing tools to understand if a message is derived from a chatbot in Russia, for example, as opposed to a human. The need and space for the ‘human in the loop’, the ‘human on the loop’ and the ‘human out of the loop’ was debated, in terms of where AI boundaries are enabled and controlled (Leins and Kaspersen 2021). Essentially these terms determine the extent to which computers versus humans exercise control. For example, is the human active within the process (human in the loop), a supervisor within the process (human on the loop) or is the AI in control with processes for explainability in place. In addition, the need to ‘bind’ or keep connected the components that manifest the record was discussed. The different rates of ‘degradation’ for components were highlighted, including considerations around expiration of, for example, e-signatures and e-seals.

It was notable that whilst the intention had been to mix people from across different areas of professional practice and academic discipline, this had been based on current roles and in fact a number of people had quite complicated backgrounds and areas of expertise. As such, Group 1 had more participants where records management and archival science had been particularly influential whereas Group 3 had more ICT focused participants.

Based on these discussions, three very different visions of the nature of a record were captured in an infographic (Record DNA). These can be summarised as:

- Vision One—a diplomatic view of the record with many granular components;
- Vision Two—a content/information/message orientated vision of the record with the components and systems that manifest the critical content/information/message as background;
- Vision Three—a new vision of the record with complex considerations sitting around the realities and unknowns of new systems delivery.

These were aimed at enabling professionals and students to share multidisciplinary perspectives and spark debate, discussion and evolutions to support further research and new processes for maintaining the digital evidence base through time.

### The ‘ideal’ digital evidence base

During the discussions, which accompanied the activities, many critical questions were asked about the components of a record and the range of evidential needs, and whether or not our laws, policies or evidential processes to support the maintenance of the evidence base need to be changed. Within this context, the range of stakeholders were discussed and the structures and power balances. Who creates and controls records, was seen to need to shift. The ability to co-create and maintain records in new spaces with better citizen rights was articulated. Previously administrations (public authorities and corporations) have controlled the central creation of the record and there has been a sense that that this is the official record with greater authority than a citizen’s account. These structures have helped with the exercise of a centralised power. New systems approaches allowing all parties to input into the records creation to enable an accurate and mutually accepted account of events are now possible. These ideas of participatory record creation and keeping were noted to have been increasingly adopted with success in health contexts where they have provided for a more detailed account of a patient’s health, including their mental health, and better treatment plans more suited to their personal circumstances (Coulter and Oldham 2016). These approaches have the potential to be considered more generally. In addition, the need for information/digital literacy was discussed. Whilst this has been the preserve of librarians the need to better communicate and explain in ways that are understandable to all audiences were articulated as key to the successful delivery of evidence for societal benefits. In addition, information systems approaches to information behaviours and user experience were seen to be another important dimension in terms of engaging with evidence. Critically, the need for records to better engage all stakeholders at all parts of the process were observed as essential in the context of the delivery of an ideal evidence base.

Significantly, the groups did not necessarily reach a consensus on the vision they presented; however there was agreement across all participants in terms of the nature and scale of the challenge of understanding and the need to capture and ensure a trusted digital evidence base through time, taking into account the complexity of managing all the components. The need for the creation and retention of records was agreed, with shared themes on the ‘ideal’ useable digital evidence base. These included the ability to access it and objective characteristics such as its security, privacy management, full history, knowing where all copies or components are, transparent audit trails and accessibility by all stakeholders. Audit and transparency encompassed goals around a capacity for explainability in a world of AI. The requirements for new egalitarian power structures (i.e. the need for all stakeholders to input into record creation and access to ensure accuracy of information) and human centred recordkeeping were strongly articulated (i.e. shared perspectives rather than a central bureaucracy holding its own accounts).

**Fig. 6:**
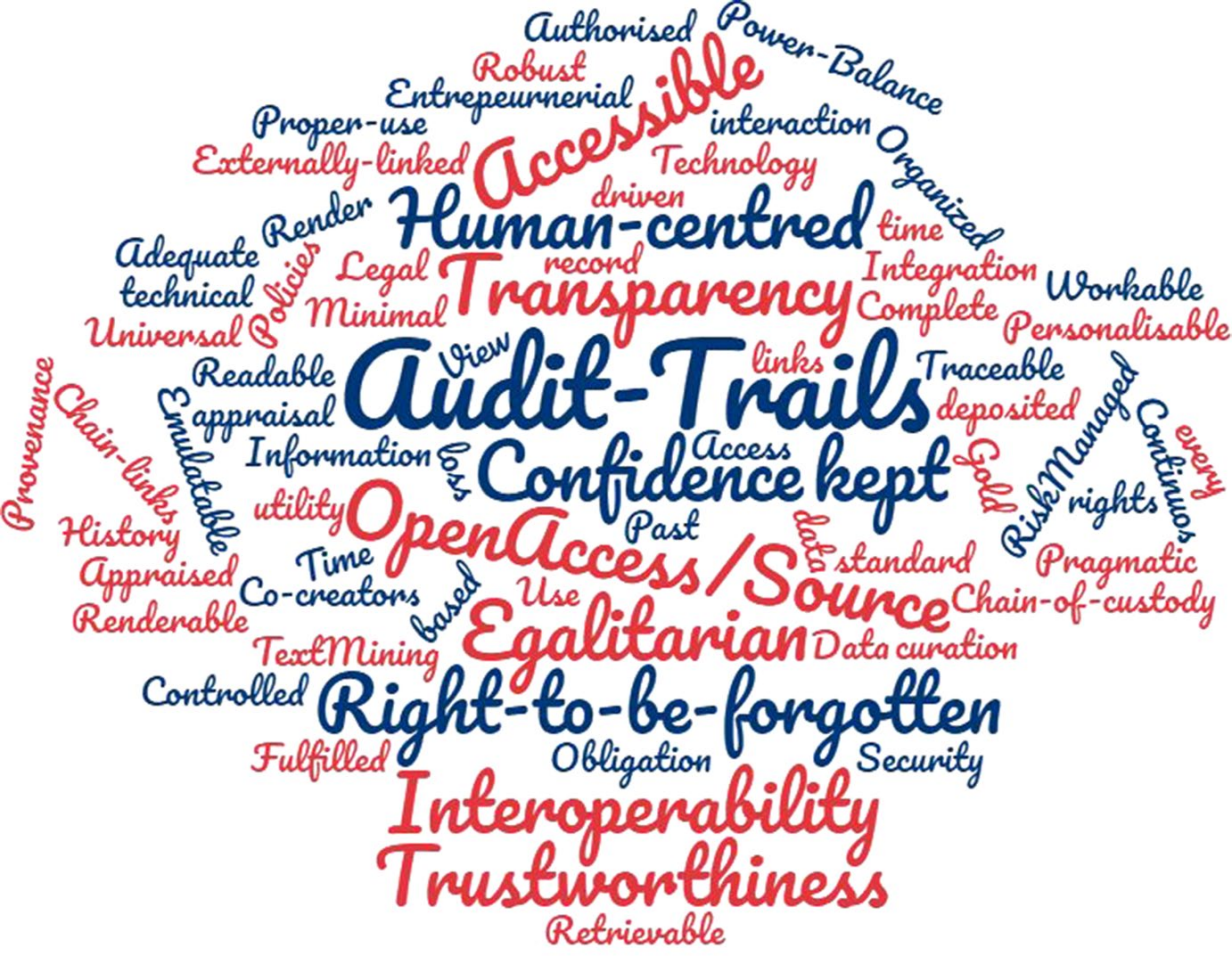
Word cloud of the most prominent elements in the visions of the ‘ideal’ digital evidence base

Interestingly, within this context was the need and challenge to delete and forget. From a professional perspective ‘pragmatism’ and ‘risk management’ were noted as being required. Figure 6 provides a word cloud of the most prominent elements of the ideal visions that emerged.

### A proactive research and development agenda to sustain the digital evidence base through time

Almost 100 research questions or problems were identified as needing to be answered, or solutions found, if society is to capture and sustain the ‘ideal’ useable digital evidence base. They fall into four themes: people issues, technology, policy and processes, as well as ethics, rights and legal issues.

The wide range of questions associated with each theme demonstrates the scale of the challenge we face. For example, people questions ranged from when do people feel the need for evidence, to what ‘evidence’ is out of scope (e.g. hidden domains such as the dark web and surveillance) and why, and information/digital literacy skills gaps for professionals and user groups. Technology questions ranged from how to encode more than we currently encode and how to develop better interfaces and tools for interrogating the future digital evidence base (including how to produce the authentic experience in a virtual reality environment) to ones about what AI should do versus humans, and how to keep users engaged and inspired when algorithms are doing so much for them. Policy and processes questions ranged from what is content and what is the effect of changing purposes and/or values over time, to can ethnographic studies of what people enjoy assist with archival delivery? Ethics, rights and legal issues included understanding conflicts and boundaries between, for example, public and private uses for records, how to manage the complexities of ownership over time, how to implement data protection practice into digital records management/curation and how we will manage the right to be forgotten. The moral as opposed to the legal requirements for dictating information structures and needs were key discussion points throughout the research.

**Fig. 7:**
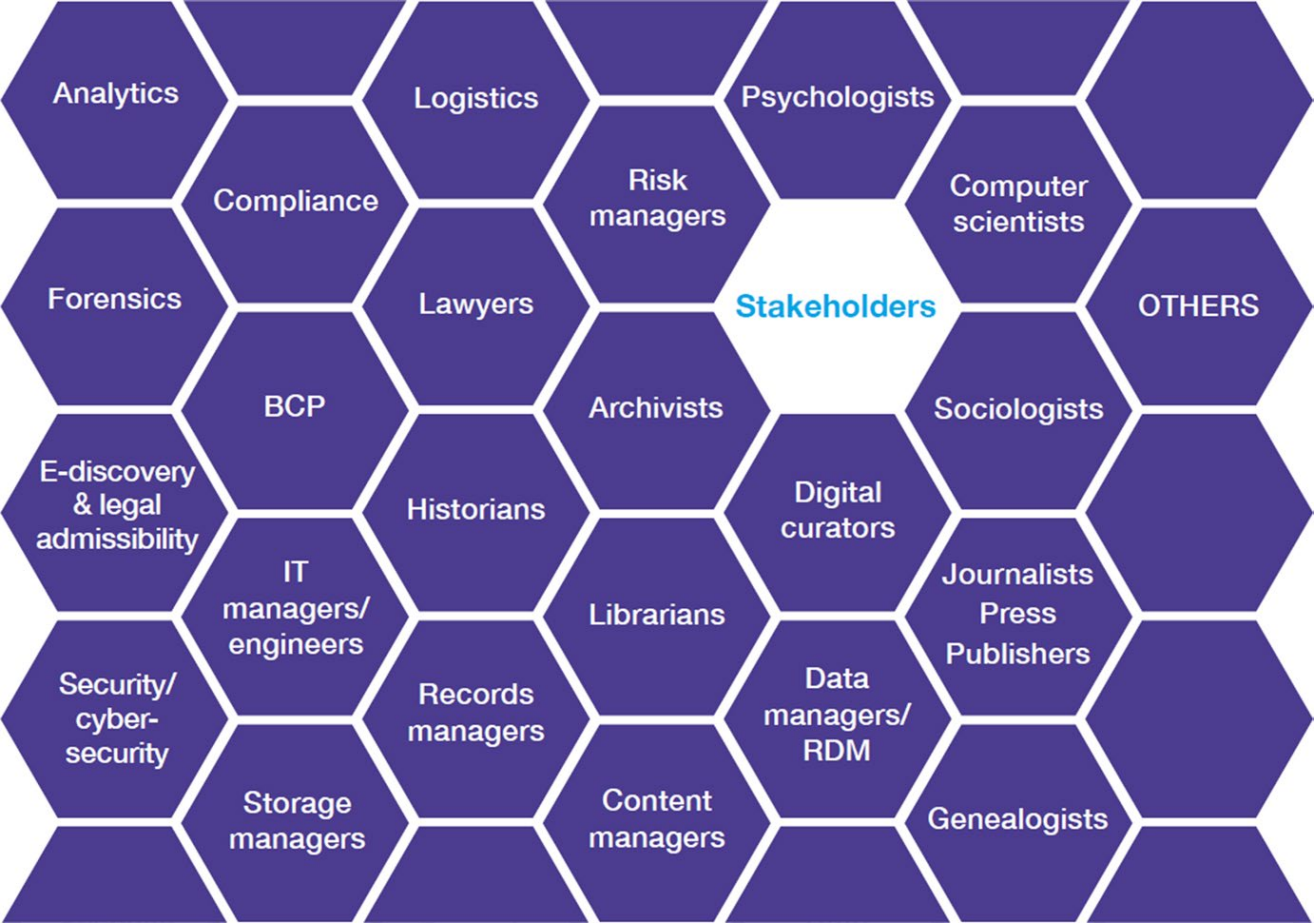
Some of the stakeholders involved in securing the ‘ideal’ digital evidence base

The questions identified as most urgent or interesting to address (Table 3) were further developed by the workshop participants by identifying specific objectives together with the disciplines and domain knowledge, skills and roles that would most likely be needed to undertake the work to address these challenges and problems. These included anthropologists, archival scientists, computer and data scientists, cyber security, digital forensics, economists, educators, historians, lawyers, librarians, philosophers, records managements and statisticians and users/stakeholders (see Fig. 7). However, it was noted that many backgrounds were needed and that the questions, challenges and problems should be opened up as widely as possible. In addition, it was deemed to be important to understand and involve stakeholders in this process to develop understanding of evidence needs and to better facilitate human-centred recordkeeping as well as transparency and accountability.

**Table 3:** Research questions identified as being the most urgent or interesting to address to deliver the useable digital evidence base

Research question/practical problem
1. What are the ethical implications of the archivist as an access mediator in a digital world?
2. What are the mutual relationships that support trustworthiness among archival institutions creators and users/consumers?
3. How do we implement appropriate data protection policies and practices into digital RM?
4. How can we best access information through time (in an affordable manner)?
5. What can we gain from applying LIS information literacy frameworks, including digital literacy and media literacy, into archival science education and practice?
6. How do we present ‘technical proofs’ to humans to get humans to trust machine/AI? Which attributes do people trust and how does this vary with beliefs/culture? Where is the human in the loop?
7. What is the philosophy of evidence in digital records? How do we understand the existing cultural and societal use of ‘evidence’ across international and domain boundaries?
8. How might psychological/behavioural characteristics of the different roles in the management of digital records affect achieving the ideal digital evidence base?
9. How do we design the archive of the deleted, the missing and the unknown?
10. How can we manage the problem and scale of managing, preserving and using data and records which are now generated/created in quantities beyond human capabilities?

An attempt was made to map these questions/practical problems to the Cynefin framework (Snowden 2010), which helps to make sense of problems and situations in different contexts in order to determine appropriate action. Problems characterised as falling into the framework’s ‘simple’ domain, often have a single one right answer and call for ‘best practice’ action; those in the ‘complicated’ domain, where there may be more than one ‘right’ answer, call for a ‘good practice’ response. For problems falling into the ‘complex’ domain an answer or solution may exist which is not currently known, calling for ‘emergent practice’ and research; and problems falling in the domain of ‘chaos’, where there is no solution or right answer, call for ‘novel practice’. Some of the research questions and practical problems in Table 3 have the characteristics of the Cynefin framework’s ‘complicated’ domain. For example, including additional information literacy education (problem 5, Table 3) that engages children from an earlier age with the complexities of navigating the digital evidence base in order to introduce critical thinking more automatically. There is a complexity in this in terms of getting the educational programmes right and evolving them through time. One initial educational tool was developed as part of the Record DNA research network’s output, a map enabling young people to consider how to better manage their digital footprint (Record DNA). Another potentially complicated domain problem is the implementation of appropriate data protection policies and practices into digital records management (research question 3, Table 3), which is achievable through (good practice) navigation by experts. However, managing personal data are not always straightforward; for example, as AI evolves if the law does not evolve at the same pace ‘emergent practice’ (the unknown solution response to a complex problem) may be needed.

The results of the wider survey about the relative importance of the research themes were inconclusive. Only two issues scored above four on the five-point scale (i.e. were considered to be a high priority with relevance across disciplines and/or domains of practice), both of which have high level policy and legislative dimensions. These were i) personal data management and privacy issues, including how to implement data protection practice into digital records management/curation, how to manage the right to be forgotten, how might attitudes to privacy change and the actual possibility of anonymisation; and ii) legislative domains, including understanding the existing use of “evidence” internationally, across sectors and culturally, and the ability of access systems to provide retrospective juridical contexts and social contexts. The priorities may have been partly influenced by current challenges at the time, in particular GDPR, Brexit, ongoing issues about privacy and access to information (e.g. in the big data context and security). For full details see Record DNA.

The case for a research and development agenda was set out in an infographic aimed at funders, academics, practitioners and others interested in forming collaborative partnerships to tackle the challenge of sustaining the digital evidence base through time (see Record DNA). The agenda will naturally evolve over time as new technologies, and consequent challenges and opportunities, emerge, and it will require many stakeholders, as outlined in Fig. 7.

## Discussion of key insights

Just as existing ‘definitions’ and perspectives of a digital record in the literature vary so did the perspectives and understandings of a record’s DNA within the research findings. This aligns to Lemieux’s assertion that, “there is no one true conceptualization of the record… but many different conceptualizations” (2001, p 82) and as Yeo notes it is not surprising that perspectives are influenced by disciplinary and professional backgrounds in terms of understanding and interacting with records (2007, p 316) which as such present different viewpoints. Clearly the prior discourses that existed, influenced or mirrored some participants’ perspectives and contributions. Some reflected the diplomatics view (Duranti 1989a, b and 2010; Voutssas 2017), others a focus on the logical and material characteristics of the digital (e.g. Bearman 1996; Upward 1996; Thibodeau 2002). For some, the vision of a record was entirely transformed (as in Fig. 5) capturing the dynamic, liquid and flexible digital world with ‘recordness’ judged by the user for whatever purpose, in whatever context, time and space. For others ‘something’ digital not intended as a record will become a record and, as such, in Fig. 4 it is the ‘information’ that was the central focus with the processes sitting around this. In Fig. 3 ‘content’ was mapped into a process and societal context. These latter perspectives align to ideas of “the ever-broadening layers of infrastructure and context through which digital records move” (Acker 2017 p 316). The large number of DNA components, the granularity, reflects a level of complexity that suggests the nature of a digital record could align with Smit’s application of Morton’s concept of a ‘hyperobject’. However, it is important to note that Smit further asserts that “[G]etting a grip on a hyperobject is a self-contradictory goal” (Smit 2017 p 261). The need for a 3-dimensional model of a record’s DNA was expressed. This is similar to the call for the records continuum model needing a 3-dimensional representation (Reed 2005) and Brothman’s proposal that “a helical model best meets the challenges of visualizing and representing the temporal complexities of record-keeping” (Brothman 2006, p 238). An emergent finding was the idea of encoding to bridge levels of abstraction, disciplines and uses taking in the intrinsic and extrinsic qualities of the record.

In all of the discussions, the role of the individual, society, trust, laws and standards were key. These were seen both to influence and to be integral to the actual record. Access, parity of access and rights have become critical questions and include considerations of record creation and use. For the majority, the recognition of social context as an element of a record’s being was evident. This harkens back to the work of McKemmish and the way in which a record is forever in a “process of becoming” due to the contextual situation (1994 p 200). In this current study the content and structure were not seen as fixed but also influenced and changed by these contexts. The context was many layered needing to be read in terms of the creator(s) and subsequent user(s) positionality and approach to the content, with physical and cultural settings and backgrounds positioning the information. This included considering information behaviours (Wilson 1999) and user experiences (Beauregard and Corriveau 2007) taking into account the transition of content or information through cultural settings as well as devices and technologies. There are questions as to whether record DNA is contingent on context; how much is exante and expost, and what does need to be captured in terms of the cultural value and qualities of a physical object when digitised. Discussions included thinking about how content is and will be navigated and explained in an AI/machine learning world, where users are not necessarily privy to each step of the creation process nor always able to easily understand these processes, in line with Bunn (2020). An important aspect of this, was in terms of questioning how all processes support the production of truth. The need for content with data quality, reliability and truthful trusted production, as opposed to the production of ‘flawed’ records, was highlighted with the dangers of ‘fairwashing’ highlighted. This is a conversation that has been growing in the technology sphere in terms of the risks of rationalising the use and explanations of the applications of AI/algorithms (Aïvodji et al. 2019). For some, the provenance, including data provenance, and chain of custody or data lineage were the imperative elements to deliver recordness. These are the concerns of the data scientist in terms of determining data governance, provenance and lineage (see https://bi-insider.com/posts/data-lineage-and-data-provenance/). All of the discussions highlighted the place of the ‘human in the loop’ and the professions sitting around production. Many wanted the ‘human in the loop’ as far as was possible whilst others saw a greater likelihood and potential of the ‘human on the loop’. In both scenarios a range of knowledge professionals are needed, albeit professional boundaries have blurred custodians and mediators, resulting in overlapping competencies and responsibilities, e.g. in terms of the roles of archivists, computer scientists, digital curators, information and records managers, lawyers and librarians. Information literacy needs were emphasised but wider forms of literacy too in terms of a range of technical, legislative and evidential literacy capabilities, such as the ability to understand an algorithm, as well as a person’s own wider contextual relationship with the ‘record’ including the impact of ‘memory’ (Marsh and Yang 2017). In a digital context, memory was perceived to be conveyed in both human contexts and digital forms, including traces and understandings from the record as held or used in a particular device. The need for technical masterclasses with very wide-ranging components, from philosophical to coding considerations, was discussed.

Key in the conversation was a focus on processes. ‘Production’ and ‘creation’ were more commonly expressed terms for record delivery rather than action and transactionality. Transactionality was perceived by some to be valuable in organisational records management but potentially not to be sufficiently inclusive. Time was an important element with production occurring through multiple processes rather than a single transactional point. Within the production were fundamental questions about the role of humanity in record creation. For example, which parts of history will a machine be not only providing evidence of but also interpreting and presenting that evidence.

For many, risk rather than certainty was very much the process focus in terms of navigating recordness. The complexity of record production seemed to best align to understanding this through Acker’s lens whereby “tracing evidence through infrastructure proves to be a way to get at record-ness without getting caught up in the rigmarole of asking what a record should be or how complete it is every time we encounter “new” digital traces and unknown systems from new platforms, to emergent ways of communicating with new formats” (Acker 2017, p 316). It then places an emphasis on policies and procedures in line with Ketelaar’s (Glaudemans et al. 2017b, p 303) view that it is not archival theory which defines a record but policy. In the dynamic, liquid and flexible digital world this raises the question of whether we should focus on managing the DNA components so that “recordness” can be judged by the user for whatever purpose, in whatever context, time and space, paralleling Smit’s (2017) ‘self-authenticating’ hyperobject. If so, we should recognise the record components (its DNA) and focus on enabling the management of those granular bits through time by harnessing the knowledge and skills of many disciplines, recognising in computational archival science the need to draw in others. The delivery of ideal evidence base visions offers the chance to challenge prior assumptions and to debate the requirements across domains. Potentially to achieve this we do need changes to our laws, policies and evidential processes.

In regards to legislation, as Livelton (1996) notes, the narrowly focused perceptions of legislators are often problematic for recordkeeping professionals, since these perceptions necessarily underlie the definitions of records found in laws and statutes in particular jurisdictions. Where such definitions exist, professionals are constrained by them in their daily work, but “need not feel obliged to accept them as the sole foundation of their thinking” (Livelton 1996, p.4). Equally for some, these are valuable and it is the lack of such definitions and evidential requirements for the majority of records that presented problems in framing DNA elements.

Within the context of the ideal evidence base from an archival perspective, the issues of what does get preserved through time, as opposed to how that occurs, harken back to Bailey’s question on why not keep everything (Bailey 2008). The value and opportunities for archival data acquisition and use were seen as having shifted in the digital era. Silence, marginalisation and power were noted as critical needs to be addressed within the research agenda with new opportunities to rebalance and co-create. An example given was of the Stolen Generations in Australia who rely on archives for identity, memory, accountability, redress and recovery needs but who have had little opportunity to co-create their own evidence and narratives (Evans et al. 2015). Within this context, it then becomes necessary to build not only on the vision of Ketelaar about choices (Glaudemans et al. 2017b) but the qualities required to co-create the trustworthiness of records, which include characteristics such as authenticity, integrity, reliability (ISO 15489-1: 2016s 4 and s 5.2.2; ISO 30300: 2020s 3), but in addition those methods that sit around these characteristics together with bigger questions and needs. The visions presented and discussed in the network included a distillation of the broad spectrum of perspectives. In terms of understanding critical aspects of useability, functionality was identified as a key attribute. The latter can mean not necessarily fixing a record in order to better enable new forms of use. The liquid nature of the digital was embraced, as opposed to its fixity and stagnation. Issues of trust and information quality remained vital in the digital environment. Shifting power structures form part of this delivery and change. The positioning of the human in/on the loop, or rather at the centre of the evidence, co-creation, production and delivery, with explainable and auditable processes is key.

The nature of the research needed to address questions around sustaining and delivering the future digital evidence base reflected the full range of research typologies as defined by the OECD (2015) and adapted for the arts and culture by Lomas (2017) viz. *basic research* to acquire new knowledge without a specific application or use in sight; a*pplied research* directed primarily towards a specific aim or objective; and e*xperimental development* to produce new or improved products, processes, systems or experiences. The specific questions comprise three themes considered by some (e.g. McDonald 2005; McLeod et al. 2010) as fundamental for managing digital records viz. people issues, technology, policy and processes, as well as ethics, rights and legal issues. If we do not address these questions urgently we risk losing important evidence, heritage and history. We risk being unable to access, present and use the information in the ways the different stakeholders (academics, professionals, the public) need to and prefer. We risk not having the shared and mutually understood concepts and vocabulary for the multiple professions and disciplines needed to develop solutions to safeguard the digital evidence base. However, most of the questions are, perhaps unsurprisingly, not straightforward to answer or address. We can try to understand them using the Cynefin ‘sense-making’ framework to aid identification of simple, complicated and complex solutions (Snowden 2005, 2010). The majority are *complex* meaning that cause and effect can only be understood in retrospect and experimentation is needed to find answers, resulting in emergent practice. In addition, we can set out and prioritise questions as the network has sought to do. Clearly, this is a grand challenge which requires careful development and mapping.

## Conclusion

The grounded theory approach used in this research further opened up and revealed the complexities of the challenge that we face in delivering and securing a digital evidence base through time. The original premise for this research was that the digital evidence base is at risk because of the challenges we face in terms of how digital records are created and captured, how we preserve them as evidence and how we access and use them in the context of constant change. This research did not resolve this challenge but rather confirmed its significance and scale. Whilst diplomatic tools delineate the nature of the problems, as Smit (2017, p 261) points out “digital records might [be] beyond the control we were used to in the analog environment.” We would assert that digital records do now need new and novel solutions to ensure the availability of the evidence base. The work exposed the different perspectives and approaches being used across disciplines and professions to probe the issues raised. Managing the complex range of original evidence through time remains one of the biggest challenges records professionals face. It is an international challenge which cannot be solved in isolation but requires sustainable multidisciplinary collaboration to tackle it. The challenge comprises a network of problems but, in addition, there are very real opportunities to be harnessed. In a digital era, records have the potential to be co-created and better produced for all citizens, rebalancing power and establishing trust. Creating, managing and providing access to a trusted, useable ideal evidence base is, as asserted at the outset of this article, a grand challenge. In addition, it links into other grand challenges such as anthropogenic climate change. Anthropogenic climate change is a grand challenge in its own right but the better management of the digital evidence base has a role to play in this challenge as was highlighted in the research findings in terms of discussions around record retention and storage choices and power solutions, and by McLeod (2020) in the context of storage economics and trust. However, having exposed the scale of the issues, we would like to conclude by asserting the case that it requires and merits a convergence research approach.

Convergence research is generally inspired by the compelling need to address a specific challenge or opportunity, whether it arises from deep scientific questions or pressing societal needs. It requires, “deep integration across disciplines*.* As experts from different disciplines pursue common research challenges, their knowledge, theories, methods, data, research communities and languages become increasingly intermingled or integrated. New frameworks, paradigms or even disciplines can form sustained interactions across multiple communities” (National Science Foundation 2017). The differing perspectives in the work discussed in this article identified a need for increased dialogue to move us towards more shared understandings, to facilitate strategic thinking and new research, which will ultimately deliver practical solutions for securing the digital evidence base. Within the 10 themes the National Science Foundation (NSF) has focused on for convergence research is the theme of harnessing data (NSF, https://www.nsf.gov/news/special_reports/big_ideas/harnessing.jsp). However, it has taken an approach to this which is potentially positivist, calling on quantitative science with a particular engineering lens. We would argue that for true convergence theory other disciplines and wider approaches need to be taken. The questions around securing the ideal evidence base are potentially more complex than those the NSF is focusing on. In addition, they are fundamental to a functioning and positive society.

We need to develop short and longer term strategies to the production and maintenance of our digital evidence base, which will need to be tested and trialled through time, and to look at more fundamental philosophical issues. Revolution is required. Revolution in our conceptual thinking to understand the digital reality and the DNA of digital records; revolution in the language we use to communicate with the multifarious stakeholders; and revolution in our collaboration with the different disciplines that can help solve the problem. Convergence research focusing on the ideal evidence base is a critical grand challenge.
